# Pandemic amnesia: the absence of pandemic prevention and preparedness in Indonesia’s 2024 presidential election

**DOI:** 10.3389/fpubh.2025.1554289

**Published:** 2025-07-08

**Authors:** Hana Antonio, Yodi Mahendradhata

**Affiliations:** ^1^Independent Researcher, Jakarta, Indonesia; ^2^Department of Health Policy and Management, Faculty of Medicine, Public Health and Nursing, Universitas Gadjah Mada, Yogyakarta, Indonesia

**Keywords:** pandemic, preparedness, election, Indonesia, policy, health policy

## Abstract

The COVID-19 pandemic exposed profound weaknesses in global and national capacities for pandemic preparedness, emphasizing the urgent need for robust public health policies. This manuscript examines Indonesia’s 2024 presidential election, where leading candidates largely neglected pandemic prevention and preparedness despite the enduring socio-economic and health impacts of COVID-19. This work highlights the critical need to embed pandemic preparedness into electoral platforms, national policies, and global health agendas. Kingdon’s three streams framework (problem-policy-politics) illustrates how elections shape the prioritization of preparedness through shifts in political will. Therefore, public health advocates must strategically influence electoral agendas by forming unified policy proposals, developing tools like candidate scorecards, and mobilizing community education. Making pandemic preparedness a central electoral issue ensures readiness for future health crises and strengthens systemic resilience.

## 1 Introduction

The COVID-19 pandemic has profoundly impacted every sector of human life globally and locally. Lockdowns and quarantines significantly affected the economic sector, resulting in decreased consumer spending and a reduced labor supply due to employee illness or isolation. This disruption in supply and demand led to an increase in prices for certain commodities, as a result of a reduced supply and heightened demand (1, 2). Additionally, the limited economic activity resulted in lower general tax revenue, increased government spending, fiscal deficits, and public debt (1, 2).

Socially, the pandemic imposed global changes. Measures such as social distancing, mask-wearing, and frequent handwashing disrupted routine gatherings, leading to the formation of online communities and workspaces (1, 3). While online learning and remote work continue to save travel time and costs, they also resulted in the loss of vital social skills and engagement opportunities for school-aged students (1, 3). Moreover, the fear of COVID-19 transmission and its severity led to frustration, mental health issues, and unhealthy cleanliness habits among some individuals (3).

The COVID-19 pandemic thus serves as a powerful demonstration of the need for sustained, adequately resourced, and internationally coordinated pandemic preparedness efforts. Pandemic preparedness is a continuous process of planning, exercising, revising and translating into action national and sub-national pandemic preparedness and response plans (4). Member States of the World Health Organization (WHO) have agreed to a global process to draft and negotiate a convention, agreement or other international instrument under the Constitution of the World Health Organization to strengthen pandemic prevention, preparedness and response (5). The 2024 World Health Assembly (WHA) marked the adoption of Internal Health Regulations (IHR) and extended negotiations on the pandemic treaty to 2025. The underlying reason for the pandemic agreement was to ensure equity in access to the tools required to prevent pandemics and in access to healthcare services during the pandemics (6). Looking back at the COVID-19 pandemic, high-income countries (HIC) occupied most of the vaccine stocks and pharmaceuticals, leaving lower-income countries (LIC) with the leftover pieces (6). Therefore, the pandemic agreement will serve as a social bargain, safeguarding future generations from the devastating and inequitable impacts of pandemics. It aims to facilitate the open exchange of real-time scientific information and promote the equitable allocation of medical countermeasures in an interdependent manner (7, 8).

Year 2024 made history as the biggest election year, where more than 50 countries undertaken national elections, including Indonesia (9). These massive elections will significantly impact economies, international relations, public health, and pandemic prevention through policies determined by the elected leaders (9). These policies encompass broad statement of goals, objectives and means that create the framework for activity leading to implementation (10). In Indonesia, the leading presidential candidates have notably omitted pandemic preparedness from their platforms. This perspective manuscript aims to examining the absence of pandemic preparedness discourse in Indonesia’s recent presidential election and analyzing the implications for Indonesia’s public health security and resilience.

## 2 COVID-19 pandemic in Indonesia

While the World Health Organization (WHO) declared the COVID-19 outbreak a Public Health Emergency of International Concern (PHEIC) on January 30, 2020, Indonesia announced its first cases on March 2, 2020 (11, 12). The government then formed a COVID-19 task force team comprising medical specialists, epidemiologists, and government representatives (11, 12). The government implemented large-scale restrictions, public place closures, and rapid testing (11, 12). President Widodo’s administration’s initial pandemic response was slow and did not fully support global recommendations (11, 12). By the end of 2020, the government enacted COVID-19 vaccination and secured early doses of vaccines. However, during the peak of the delta variant in July 2021, vaccination coverage remained low (12). Vaccine uptake gradually increased, paralleling citizens’ trust in government policies (12). As of August 7, 2023, 74.5% of Indonesians had received the second dose, 69,243,490 the third dose, and 3,524,526 the fourth dose (9). On June 21, 2023, the pandemic era officially came to an end, and there was continued encouragement to maintain hygiene and healthy habits (12).

The pandemic brought to light deficiencies in Indonesia’s healthcare system, such as insufficient bed capacities and a shortage of healthcare workers, particularly during the delta wave when hospitals were overcrowded (13, 14). Early on, there was a poor supply of protective equipment and an unclear referral system (13, 14). Additionally, the pandemic lowered the utilization of social health insurance and referrals for non-communicable diseases from primary to secondary and tertiary health centers (15). The COVID-19 pandemic has also affected the utilization of essential health services for the general population as well as for vulnerable groups. Among the existing essential health services, NCD screening and routine treatments were among the most unmet services in the community (16).

Economically, the pandemic severely affected Indonesia (17–19). Mass social and mobility restrictions impacted the working poor in the urban and informal sectors the most. More than 2.6 million people experienced job loss, and continued instability of the job market (20). Reduced cash flow and profits forced companies to lay off employees (17–19). The exchange rate of the rupiah to USD consistently declined, and economic activity weakened, reducing state tax revenue. The government received advice to expand fiscal policy and increase public expenditure for post-pandemic preparedness when the economy started to grow in 2021 (17, 21).

Food security in Indonesia decreased as well during the pandemic (22). However, Indonesia did not make any specific food assistance school programs available to children during the pandemic. There were education programs developed by selected schools for parents focusing on the importance of providing nutritious food to their children during the pandemic. Children were requested to report their breakfast and lunch meals (e.g., photos of the foods) to their teachers. Socially, the large-scale restrictions deeply affected Indonesians, especially in regional and rural areas where routine cultural and social events are common (23, 24). A lack of digital knowledge and education disadvantaged vulnerable groups such as youth and women, reducing their opportunities to grow and secure employment during the pandemic (22, 23).

Indonesia’s pandemic-preparedness architecture evolved markedly between the 2003 severe acute respiratory syndrome (SARS) episode and the COVID-19 pandemic. During SARS, policy instruments were limited to border-temperature screening, hospital-based contact tracing, and short-term quarantine decrees issued by the Ministry of Health (25). Under President Joko Widodo, however, COVID-19 governance was anchored in Presidential Regulation No. 82/2020, which created a unified Task Force for COVID-19 Handling and National Economic Recovery, authorized emergency-budget reallocations exceeding 5% of GDP (26), and introduced tiered social-restriction regimes. The Government also rolled out the PeduliLindungi digital-tracing application (27), expanded the COVID-19 referral laboratory network from one central laboratory to 685 laboratories across 34 provinces within 12 months (28) and launched a vaccination program that delivered more than 450 million doses by January 2023 (29). Compared with South Korea’s early, high-volume testing strategy and Vietnam’s rapid border closures and centralized quarantine, Indonesia relied more on adaptive social restrictions and accelerated vaccine procurement, illustrating the diverse policy mixes through which Asia-Pacific states balanced epidemic control with economic resilience (30).

Responsibility for formulating pandemic-preparedness policy in Indonesia lies primarily with the Directorate-General of Disease Prevention and Control (DG-P2P) of the Ministry of Health, which drafts the National Health Crisis Preparedness and Response Plan in consultation with the National Disaster Management Agency (BNPB) and other line ministries. Legal authority is provided by Law No. 6/2018 on Health Quarantine, while operational leadership during health emergencies is vested in the Head of BNPB, who acts as Incident Commander of the National Task Force created under the same presidential regulation. Strategic objectives are further embedded in the National Medium-Term Development Plan (RPJMN) 2020–2024, linking health-security targets to a multisectoral disaster-risk-governance agenda (14). This arrangement reflects a “whole-of-government” model comparable to South Korea’s Central Disease Control Headquarters and Vietnam’s National Steering Committee for COVID-19 Prevention and Control, where health ministries provide technical direction under high-level inter-ministerial coordination (31, 32).

## 3 Importance of pandemic preparedness

According to the World Health Organization (WHO), pandemic preparedness is a continuous process of planning, exercising, revising, and translating national and sub-national pandemic preparedness and response plans into action (4). It is an integral part of preparedness for any health emergency, such as disease outbreaks, natural disasters, or chemical incidents, and aligns with the implementation of the International Health Regulations (2005) (4).

In April 2022, WHO released a policy brief encouraging Member States to develop an integrated approach to respiratory pathogen pandemic preparedness planning and enhance national and sub-national functional capacities for preparedness (33). WHO identified four core areas: planning and coordination, risk communications and community engagement, health intelligence, and health interventions (33). The European CDC (ECDC) suggested that countries formulate pandemic preparedness plans to strengthen existing systems, test new systems, ensure resource allocation, and sustain economic activity during pandemics (34).

Applying the abovementioned WHO four-pillar framework reveals a nuanced picture of Indonesia’s pandemic preparedness. In planning and coordination, Indonesia’s approach to coordinating COVID-19 response efforts among its diverse regions and communities involved a mix of centralized and decentralized strategies, cultural integration, community empowerment, and adaptive governance. Despite challenges in coordination and policy implementation, the use of local wisdom, technological support, and social capital were key elements in managing the pandemic effectively (35, 36). In risk communication and community engagement, inconsistent and poorly communicated social distancing policies led to public confusion, resistance, and mistrust in Indonesia (37, 38). Frequent policy changes, unclear terminology, and mixed messaging undermined public confidence in the government’s pandemic response. For health intelligence, Indonesia’s efforts to expand RT-PCR capacity and implement real-time genomic surveillance during the COVID-19 pandemic were hampered by logistical, infrastructural, and systemic challenges. Addressing these issues through improved supply chains, enhanced laboratory infrastructure, and better communication strategies was essential for an effective pandemic response (12, 39). Finally, Indonesia’s approach to managing the COVID-19 pandemic involved a combination of tiered social restrictions and mass vaccination efforts (40, 41). The effectiveness of these interventions was influenced by public trust, targeted distribution strategies, and support from religious authorities (42). Despite challenges, these measures contributed to controlling the spread of the virus and promoting public health. Notwithstanding, limited primary-care surge capacity and medical-oxygen bottlenecks exposed enduring health-system fragilities (14).

A broader cross-country perspective underscores how such strengths and gaps are shaped by the interplay of legal frameworks, surveillance infrastructure, and inter-sectoral governance. The United States relied on a federal model anchored in the National Response Framework (NRF) and the Strategic National Stockpile (SNS) to bolster state-level surge capacity during emergencies, including the COVID-19 pandemic (43, 44). The NRF provides a comprehensive guide for national disaster response, outlining scalable, flexible, and adaptable structures to coordinate roles and responsibilities across various levels of government. China, drawing on post-SARS reforms, employed a hierarchical command system that enabled rapid city-wide lockdowns, developed Fangcang shelter hospitals, and deployed population-scale digital mobility tracing (45, 46). Australia activated its 2019 Health-Sector Emergency Response Plan, leveraged the National Medical Stockpile, and pursued data-driven suppression through extensive PCR testing and targeted local restrictions (47, 48). Taiwan re-established its Central Epidemic Command Center within 24 h of the first WHO alert, integrated travel history with the national health-insurance database for real-time risk scoring, and achieved universal mask use without nationwide lockdowns (49). South Korea, informed by the 2015 MERS outbreak, amended the Infectious Disease Control and Prevention Act to permit rapid contact tracing via telecommunications data and scaled domestic RT-PCR test-kit production within weeks (50). Across these settings, clear legal mandates, high-level coordination, and sustained investment in diagnostic and digital infrastructure consistently emerge as critical pillars of effective pandemic preparedness.

## 4 Analysis of the recent presidential election

Indonesia held its presidential and legislative elections on February 10, 2024 (51). Prabowo Subianto won with 58.6% of the vote, defeating Anies Baswedan (24.9%) and Ganjar Pranowo (16%) (51). Each candidate had distinct campaign goals and political targets. Baswedan focused on financial goals, including raising the tax-to-GDP ratio to 10–16% by 2029, creating 15 million jobs, reducing annual inflation to 2–3%, minimizing staple product imports, and promoting sustainability plans like renewable energy incentives and forest rehabilitation projects (51). Subianto proposed diverse plans, including free lunch and milk during school breaks, increasing the defense budget, maintaining Indonesia’s non-aligned foreign policy, and achieving food, water, and energy self-sufficiency (52). Pranowo aimed to increase renewable energy to 30%, raise the defense budget to 1–2%, accelerate reforestation, create a tax digital collection system, and strengthen the national anti-corruption system (52).

In regard to health issues, all presidential candidates covered infectious disease issues much less than maternal and child health, health reproduction, and nutrition. Anies Baswedan, stands out for his commitment to bolstering community-based surveillance (53) In addition, Anies Baswedan and Ganjar Pranowo mentioned increasing vaccination coverage to fight outbreaks. Ganjar Pranowo additionally emphasized the importance of community volunteers in mitigating the outbreaks. Anies Baswedan additionally identified integrated information systems and community-based surveillance as key components of their pandemic preparedness plans (53). One of the top priorities for pandemic preparedness identified was the digitalization and integration of public health systems.

## 5 Absence of pandemic preparedness discourse

The General Election Commission organized three presidential and two vice presidential debates prior to the presidential election on February 14, 2024, with notable absence of a pandemic preparedness discourse (54). These debates focused on human rights, geopolitics, the economy, foreign relations, and security and defense, but did not discuss the COVID-19 pandemic or future pandemic plans (54–56). Campaign speeches and social media posts from all candidates covered healthcare services, financing, and healthcare worker shortages, but ignored pandemic preparedness (57).

Domestic and global political dynamics influenced Indonesia’s 2024 presidential election, as they did others worldwide. Political barriers to pandemic preparedness include domestic dominance, multilateral gridlock, socio-economic conditions, transnational challenges, and short-termism, with decision-makers focusing on immediate issues (58). This has led to some countries, such as Indonesia, where presidential election candidates have chosen not to address and discuss pandemic preparedness during election debates and campaigns.

## 6 Implications for public health security

Ignoring pandemic preparedness can lead to severe public health consequences (59, 60). Poorly prepared countries often face delayed reactions to pandemic threats due to factors such as devaluation, delays, denial, and distrust, which can lead to increased caseloads, mortality rates, and overwhelming healthcare systems (61, 62). Without sufficient public health infrastructure, policies, and legal frameworks, countries will struggle in future pandemics (61, 62).

Failure to adequately prepare and respond to pandemics can result in increased national health spending, social disparities leading to inequity in health services, and destabilized national finances due to the allocation of a larger portion of GDP to health services and products (61, 62).

In the United States, a country with substantial resources and developed public health infrastructure, COVID-19 caseloads and mortality rates were among the highest globally. Experts suggest that this was due to underutilized preparedness capacities and a lack of an appropriate legal framework, which contrasts with the situation in East Asian countries (63). Political leaders undermined public support for COVID-19 measures, and public health communication was inconsistent (63). Despite technological advancements, the US failed to effectively aggregate, analyze, and publish real-time COVID-19 data and genetic testing for tracking (63). Social disparities also led to inequity in testing and healthcare services for certain ethnic groups (63).

On the other hand, East Asian countries like Japan and South Korea implemented effective pandemic preparedness measures (64). They developed or improved emergency legal frameworks to clarify authority responsibilities, focused on public health institution and healthcare system preparedness, prevented in-hospital disease transmission, and ensured transparent information sharing about public health measures, including mask usage, public place closures, aggressive contact tracing, and school closures (64).

## 7 Discussion

National governments should invest in public health and social protection systems, addressing inequalities for vulnerable groups (65). All governance levels should encourage transparency and partnerships with civil society and non-government organizations (NGOs) (65). Legal instruments and recommendations for pandemic preparedness should align with the WHO’s global health recommendations (65).

The integration of public health systems that include case investigation, contact tracing, and epidemiologic analysis with upgrades in technology is also an essential part of a pandemic preparedness plan. Thus, it will enhance public trust and create more space for public health interventions in the community (66). Strengthening primary healthcare, beginning with the financing reform, will also improve routine care and the response to health emergencies.

The integration of public health systems also requires sustained high-level political leadership. Therefore, Indonesia’s political leaders need to integrate and maintain pandemic preparedness plans across all governance levels (65). Indonesia should improve public health infrastructures, capacities, and legal policies for future pandemics in tandem with its commitment to establish a financial intermediary fund (FIF) during the G20 meeting in 2022 (65).

Kingdon’s three streams (problem-policy-politics) framework (10) illuminates how political elections can influence pandemic preparedness policies. The COVID-19 pandemic heightened pandemic awareness (problem stream) and generated multiple policy solutions, such as enhanced healthcare infrastructure, stronger surveillance systems, and equitable vaccine access (policy stream). However, election results strongly shape the political stream, determining whether preparedness policies advance or stall. Thus, the election of a presidential candidate who does not prioritize pandemic preparedness can close windows of opportunity for strengthening public health preparedness policies, despite advances in the problem stream (increasing pandemic awareness) and policy stream (accumulating evidence-based pandemic preparedness recommendations). Looking back at the recent US election, Trump and Biden proposed different approaches in terms of pandemic preparedness (67). Biden requested a raise in global health’s budget for 2025 and is actively negotiating for the WHO pandemic agreement, while Trump proposed a reduced fiscal year for global health. Presidential candidates ideally should consider incorporating a pandemic preparedness plan during their election campaigns to enhance outbreak surveillance and mitigation. Thus, the challenge for public health advocates is to leverage election cycles strategically, actively aligning the problem and policy streams with supportive political environments. Doing so ensures that critical windows of opportunity for enhancing pandemic preparedness remain open and actionable, protecting populations from future pandemics. To make pandemic preparedness a central concern in future elections, public health advocates, civil society groups, researchers, and academic institutions can collaborate on three interconnected strategies. First, form a unified agenda by jointly crafting evidence-based recommendations—position statements and checklists—that candidates are asked to endorse, signaling broad consensus on health security. Second, translate data into action by creating user-friendly “scorecards” and concise policy briefs, enabling voters, journalists, and the public to objectively compare candidates’ commitments to legal, financial, and infrastructural aspects of pandemic readiness. Lastly, mobilize communities and education by hosting public seminars, academic forums, and local workshops that frame pandemic preparedness as essential to economic stability and social equity, while training grassroots leaders to highlight these issues in campaign events and debates.

Future research could begin by comparing how pandemic preparedness platforms vary across diverse electoral contexts, examining the political factors that promote or sideline health security agendas. Concurrently, studies on media framing could reveal best practices for converting technical health data into compelling election messages. Researchers might also investigate how civic, religious, and academic groups collaborate effectively to shape candidate commitments and assess whether local engagements prompt measurable improvements in campaign pledges. Testing standardized “pandemic readiness scorecards” would help clarify how voter-friendly tools sway public opinion and prompt stronger policy commitments. Longitudinal follow-up studies could then track how officials enact pandemic promises once in office, identifying systemic enablers and barriers.

National and subnational policies must integrate pandemic preparedness. Thus, pandemic preparedness should be a central topic in electoral campaigns, debates, and media briefings, reflecting political leaders’ commitment to future pandemic planning. Policymakers and future electoral candidates need to prioritize pandemic prevention and preparedness. A resilient public health infrastructure and broad public health capacity are essential to protect against future health crises.

## Data Availability

The original contributions presented in the study are included in the article/supplementary material, further inquiries can be directed to the corresponding author.
